# Titanium anodizing in a choline dihydrogencitrate salt–oxalic acid deep eutectic solvent: a step towards green chemistry in surface finishing of titanium and its alloys

**DOI:** 10.1039/d1ra01655e

**Published:** 2021-06-14

**Authors:** Juliusz Winiarski, Anna Niciejewska, Monika Górnik, Jakub Jakubowski, Włodzimierz Tylus, Bogdan Szczygieł

**Affiliations:** Group of Surface Technology, Department of Advanced Material Technologies, Faculty of Chemistry, Wrocław University of Science and Technology Wybrzeże Wyspiańskiego 27 50-370 Wrocław Poland juliusz.winiarski@pwr.edu.pl +48-713280425 +48-713203193; Technolutions Miłosz Czajkowski Otolice 38, 99-400 Łowicz Poland

## Abstract

Deep Eutectic Solvents (DESs) are “green” competitors for some conventional plating baths and electrolytes used for surface modification. Their use allows a material to be obtained with a structure different from that observed in conventional plating or finishing technologies. In this work the titanium anodizing process was investigated in a bath based on a choline dihydrogencitrate salt and oxalic acid (1 : 1 molar ratio) green solvent. Titanium anodized at the lowest voltage applied (10 V) was a deep yellow color, which turned to deep blue at 30 V. The surface morphology and topography of titanium, both anodized and untreated, were monitored by optical, scanning electron (SEM and HR-SEM) and atomic force (AFM) microscopy. Anodizing at 10 V produced a fine granular morphology of the oxide layer, while anodizing at 30 V led to the formation of a probably thicker and quite uneven oxide layer, characterized by a distinct and coarse granular morphology. The average size of the micro-nodules was higher than those at 10 V and porous structures have been also identified. According to X-ray photoelectron spectroscopy (XPS) the stoichiometric TiO_2_, regardless of the applied voltage during anodizing, was practically the only component of the oxide layer produced on titanium in the DES bath. At 10 V, the oxide layer was thicker (>10 nm) than the natural Ti passive layer (approx. 2.2 nm), which, apart from TiO_2_, also contained oxides of titanium at lower oxidation states, *i.e.* +2 and +3. Moreover, the XPS technique was supported by electrochemical impedance spectroscopy (EIS), especially in the context of the structure of the oxide layer and its interaction with a corrosive environment. The corrosion resistance of anodized titanium was assessed in 0.05 mol dm^−3^ solution of NaCl by the linear polarization resistance (LPR) technique and polarization curves. During interpretation of the impedance spectra, the layers produced by the anodizing process were described using the two-layer model. It was assumed that the inner layer formed directly on the surface of metallic titanium was responsible for the barrier properties (resistance of 2.8 MΩ cm^2^). The porous outer layer formed on it has a much lower corrosion resistance, *i.e.* 800–1300 Ω cm^2^.

## Abstract 2

Fabrication of nanometric color TiO_2_ layers through polarization of titanium in a choline dihydrogencitrate–oxalic acid DES anodizing bath.

## Graphical abstract

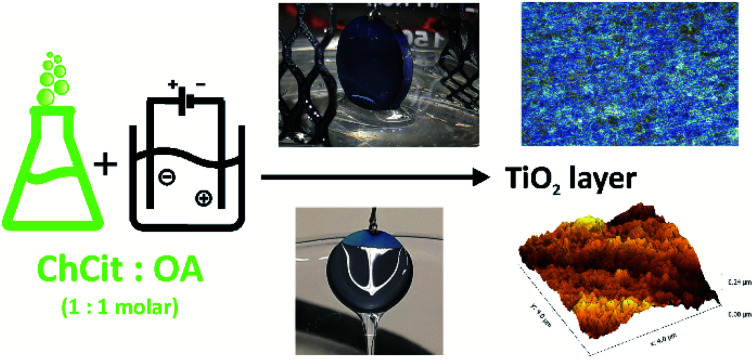

## 1. Introduction

Titanium is a metal with very good corrosion resistance and has a very high strength-to-density ratio. These properties determine the wide use of this metal for the production of many devices operated under aggressive conditions. The corrosion resistance of titanium is due to the passive oxide layer spontaneously formed on its surface, the thickness of which can be up to 10 nm.

The morphology and structure of the oxide layer can be modified and the TiO_2_ layer thickened by an electrochemical or chemical process. The titanium anodizing process can be carried out in various solutions, among which most often these are based on: sulfuric(vi) acid, chromic(vi) acid or phosphoric(v) acid, in which oxide layers are formed of relatively high roughness. Thinner and less porous passive layers are obtained by anodizing titanium in solutions such as: citric acid, tartaric acid or boric acid, and quite recently also in a deep eutectic solvents (DES). Anodizing greatly improves the corrosion resistance of titanium. Depending on the anodizing conditions, *i.e.* applied voltage, bath composition, temperature and process time – oxide layers with a thickness of several to several hundred nanometers can be obtained. Layers of different thicknesses have a different color: gold, purple, blue, orange, green. It is used in the jewelery industry to obtain decorative effects and improve the aesthetics of products, and in other industries for marking and identifying products. Titanium is used in medicine as the basic metallic material for implants. This use, in addition to corrosion resistance in the environment of physiological fluids, requires titanium to be biocompatible and bioactivity allowing the implant surface to connect to the bone. Titanium anodizing leads to an improvement in abrasion resistance, which is particularly important in the case of products manufactured for the needs of the aviation, military and tool industries. The TiO_2_ layer produced in the anodizing process can improve the adhesion of paint coatings to the substrate. Finally, titanium oxide layers with a crystalline structure show photocatalytic and hydrophobic properties, which can be used in the production of photovoltaic cells, anodes in lithium-ion batteries and self-cleaning materials.

The interest in the use of deep eutectic solvents in surface finishing of various construction metals and their alloys is constantly growing. DES are strong competitors for some conventional plating baths and electrolytes used for surface modification. Compared to standard baths, which are often corrosive and harmful to humans, DES are environmentally friendly, non-toxic, biodegradable, inexpensive and easy to produce.^1^ Can be treated as green solvents. Their use allows to obtain a material with a structure different from that observed in conventional plating or finishing technologies. In recent years it has been show that in many areas it is possible to transfer the electroplating processes from aqueous baths to those based on DES. This is the case *i.a.* with Ni and composite Ni plating,^2–5^ Zn^6^ and Zn alloy plating^7^ and especially Cr(iii) plating.^8,9^ Besides, it can be clearly seen in scientific literature that research is often directed towards surface finishing of specific construction materials such as stainless steels and light metals – titanium and aluminum alloys. For these materials, the emphasis is on the use of DES-based baths mostly for two different processes: anodizing or electropolishing.

The electropolishing of stainless steel and pure metals in the most popular DES bath based on choline chloride and ethylene glycol was studied by: Abbott *et al.*,^10^ Karim *et al.*^11^ and Protsenko *et al.*^12^ Similar DES was used in studies of aluminum electropolishing by A. Kityk *et al.*^13^ and T. M. Abdel-Fattah *et al.*^14^ Recently, A. Kityk *et al.*^15^ and W. O. Karim *et al.*^16^ investigated the electropolishing process in baths based on DES – first for Al–Mg alloy and second for titanium. Both achieved a significant reduction in roughness of the tested materials. However, there are very little works that describe titanium anodizing in DES.

In contrast to the electropolishing process, obtaining an anodizing effect of titanium in DES requires a slightly different approach. For example, it is imperative that the rate of dissolving the metal should not exceed that of forming stable oxide layer. Again, these green solvents (DES) potentially offer new environment to conduct the anodizing process of titanium, because the vast majority of titanium anodizing processes in common aqueous baths unfortunately require the use of: sulfuric acid,^17^ phosphoric acid,^18^ often with the addition of hydrofluoric acid^19^ or ammonium fluoride^20,21^ and still hexavalent chromium compounds – chromic acid.^22^ Not so long ago, C. Y. Chen *et al.* in their studies of the titanium anodizing process in NH_4_F–glycerol electrolyte showed that the addition of DES (1 : 1 molar ratio of succinic acid and choline chloride) to anodizing bath allows to control the structure of TiO_2_ nanotubes formed on its surface.^23^ This only confirms the high potential of DES liquids in the surface finishing of titanium and its alloys.

Another obvious step would be to completely transfer the titanium anodizing process to the non-aqueous DES bath. It would require the use of DES solvent, whose physicochemical properties will allow for the anodizing process in a wide range of parameters, and by selecting the current–voltage conditions one could control the thickness and structure of the forming oxide layer. The literature cited above, as well as preliminary studies, showed that the most popular DES based on choline chloride (conventionally “ChCl”) and ethylene glycol or carboxylic acids, during anodic polarization interact too aggressively not only with the titanium surface, but also with materials such as aluminum or even stainless steels.^24^ The presence of chloride ions in DES bath may be a very likely cause of this behavior. Therefore, in this work we have performed thorough research on the feasibility of Grade 2 titanium anodizing in a deep eutectic solvent based on choline dihydrogencitrate salt (conventionally “ChCit”) and oxalic acid (conventionally “OA”) in 1 : 1 molar ratio. Looking from the point of view of electroplating and anodic polarization of metals in the DES baths, ChCit is not as popular hydrogen bond acceptor as choline chloride. ChCit finds application in biotechnology as the component of *i.a.* enzyme-friendly DES solvents for biocatalysis,^25^ DES-based starch plasticizers^26^ and aqueous two-phase systems with DES solvents.^27^ In addition, successful attempts to copper electrodeposition can also be found in DES plating baths composed of ChCit and ethylene glycol.^28^ Finally, thanks to the use of ChCit : OA solvent, instead of ChCl : OA one, we get more soft, *i.e.* relatively viscous and with lower ion mobility, eutectic mixture containing neither ethylene nor propylene glycol.

Anodizing of Grade 2 titanium was performed under potentiostatic polarization, and the effect of the process on the metal surface was assessed. Surface morphology, as a function of anodizing voltage, was monitored by optical, scanning electron (SEM) and atomic force (AFM) microscopy. Particular attention was paid to the structure, morphology and chemical composition of the obtained oxide layer. Due to the expected low thickness of this layer, X-ray photoelectron spectroscopy (XPS) was chosen for this purpose. The XPS technique was supported by electrochemical impedance spectroscopy (EIS), especially in the context of the structure of the oxide layer and its interaction with a corrosive environment. The corrosion resistance of anodized titanium was assessed in 0.05 mol dm^−3^ solution of NaCl also by linear polarization resistance (LPR) technique and polarization curves.

## 2. Experimental section

### 2.1. Materials and methods

The base material were disks of technical titanium (Grade 2, purchased from WOLFTEN Sp. z o.o., Poland), 14.8 mm diameter and 2 mm thick, geometric area 4.3 cm^2^, in the as-delivered form, *i.e.* without additional grinding and polishing. Before anodic polarization, titanium disks were first ultrasonically degreased in methanol at 25 °C for 10 min. Titanium was then etched and activated at room temperature in an aqueous mixture of HNO_3_ (10%) and HF (1%) for 10 s under vigorous stirring. After activation it was thoroughly washed in deionised water under ultrasonic conditions and immediately dried with compressed air to get rid of residual water.

DES bath was prepared by mixing the choline dihydrogencitrate salt (≥98%, Sigma-Aldrich) with oxalic acid dihydrate (ReagentPlus®, ≥99.0% (GC), Sigma-Aldrich), both as supplied, in 1 : 1 molar ratio at 60 °C until homogeneous and transparent liquid was formed. At this temperature the conductivity of DES bath amounted to 0.32 mS cm^−1^. The electrolyzer for anodizing consisted of two rectangular mixed metal oxide titanium cathodes (MMO 167 by Umicore, N-type mesh) with 6 cm × 3 cm dimensions placed in parallel in a 250 ml thermostated electrochemical cell. Titanium disk has been placed vertically between two cathodes with 20 mm spacing on each side, and the current was supplied to anode with a 304 stainless steel wire with a diameter of 0.5 mm (the steel did not come into contact with the bath during anodizing). Plating bath was not mechanically stirred. Anodic polarization was realized at a constant voltage *U* = 10–30 V for 10 min at 60 °C. Then the samples were thoroughly rinsed in deionised water (ultrasonic assisted), rinsed in methanol (also ultrasonic assisted), vacuum-dried and stored in a desiccator under argon atmosphere until required.

### 2.2. Research techniques

The basic tool for investigation of the surface morphology of anodized titanium was Leica DM6 M upright materials microscope equipped with Leica Flexacam C1 digital camera. The whole set worked under the control of Leica Application Suite X (LAS X) software. The observation of the samples was performed by using LED illumination and incident light contrast methods: Brightfield (BF), Darkfield (DF), Differential Interference Contrast (DIC) and Polarization (POL). The images were captured at approximately 500× magnification in 12 Mpix resolution, using Differential Interference Contrast (DIC) and *Z*-axis stitching technique to obtain extended depth of field.

FEI Quanta 250 SEM microscope equipped with SDD Octane Elect Plus EDS detector was used to evaluate the effect of anodic polarization. SEM images have been captured in SE (secondary electron) mode with accelerating voltage of 10 kV, under 10^−4^ Pa pressure, sample tilt of *ca.* 60 °C and dynamic focus. EDS analysis was performed at take-off angle equal to 37° within the area of *ca.* 100 × 100 μm at 25 kV accelerating voltage. SEM/Ga-FIB FEI Helios NanoLab™ 600i microscope was used for high resolution imaging of the surface. Surface topography was characterized by Nanosurf FLEX-Axiom atomic force microscope (AFM). Contact mode was used (static force) with a CONTR bar, while the measurement parameters were as follows: area 4 μm × 4 μm, 1024 points per line, 1.5 s per line, 20 nN force.

The chemical analysis of titanium surface before and after anodic polarization in DES bath was performed by X-ray photoelectron spectroscopy (XPS) using a SPECS PHOIBOS 100 spectrometer equipped with a non-monochromatized Al source (1486.7 eV). The spectrometer operated at 250 W for high resolution spectra. Surface etching during XPS measurements was carried out by Ar^+^ sputtering with the beam energy of 4 keV and a beam current density of 7.5 μA cm^−2^. The spectra were processed and fitted in SPECLAB software using a Gaussian–Lorentzian curve profile and a Shirley baseline. The C 1s peak at 284.8 eV was used as the reference.

Linear polarization resistance (LPR) has been used to track the corrosion resistance of anodized titanium in deaerated 0.05 mol dm^−3^ solution of NaCl over 48 h. LPR was performed by polarizing the samples from −15 to +15 mV *vs.* open circuit potential (*E*_OC_) with a scan rate of 0.125 mV s^−1^. Impedance spectra were recorded at the open circuit potential (*E*_OC_) during 48 h of exposure to NaCl solution with a resolution of 10 pts per dec, in a frequency range from 100 kHz to 10 mHz and ac signal of 10 mV (rms). The measurements were carried out in 400 ml corrosion cell (Metrohm-Autolab) using Reference 600 (Gamry) potentiostat/galvanostat/ZRA. A stainless steel rod (geometric area exposed to the solution: 4 cm^2^) and the Ag|AgCl (3 M KCl) electrode (Metrohm) mounted in a Luggin capillary were used as the counter and reference electrodes, respectively. The geometric area of working electrode exposed to NaCl solution was 1.0 cm^2^. The corrosion cell was kept in a Faraday cage during EIS experiment. The NaCl solution was deaerated for each sample by purging with 99.9% pure argon for 20 minutes. Equivalent circuit modeling, graphing, analysis of impedance spectra, and determination of polarization resistance were performed using EchemAnalyst (Gamry) software.

## 3. Results and discussion

### 3.1. The effect of titanium anodizing on its surface morphology

**Fig. 1:**
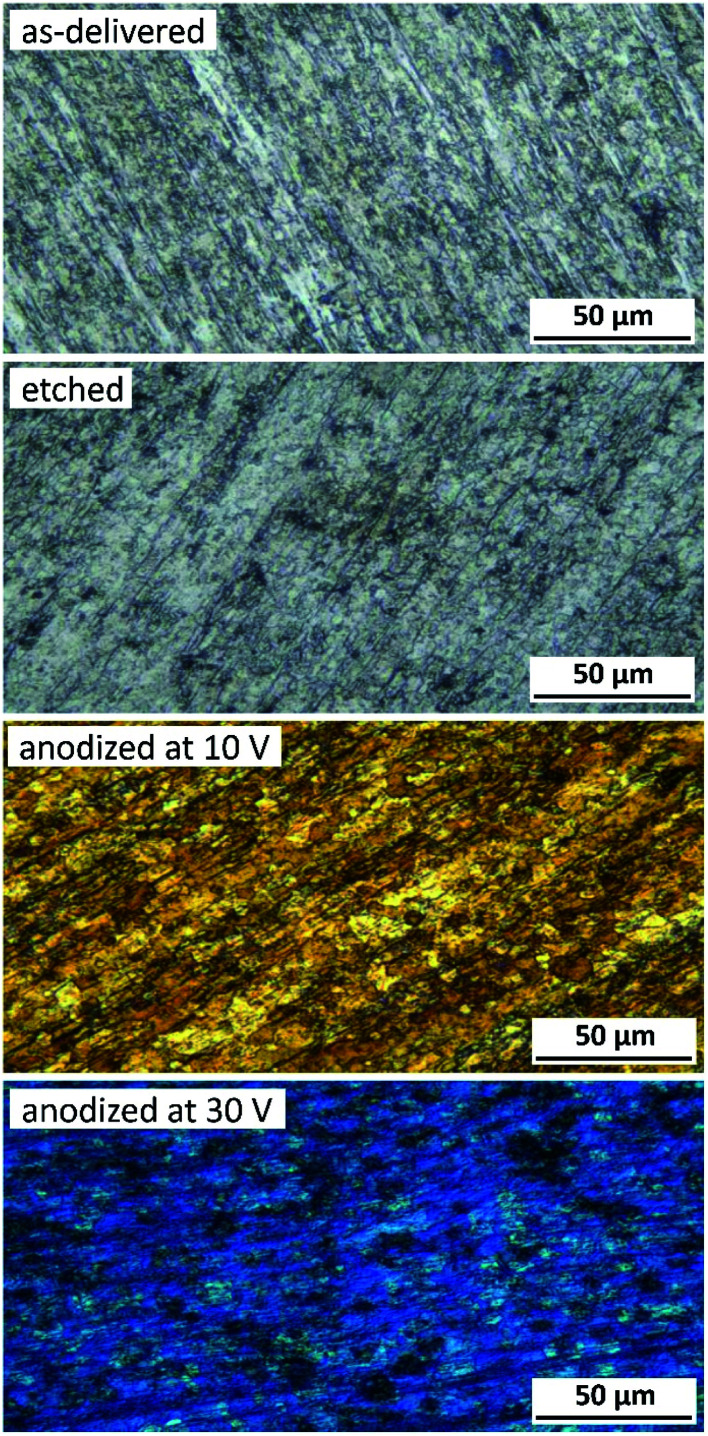
Surface morphology of titanium samples after: degreasing, degreasing and etching and after anodic polarization in a DES bath at 10 and 30 V for 10 min at 60 °C. Photographs were registered in a Nomarski interference contrast mode.

Anodizing of titanium is associated with the formation of an oxide layer, the color of which depends on its thickness, morphology and structure. Therefore, the first tool to evaluate an anodized surface was optical microscopy. Fig. 1 shows, besides a blank reference titanium surface, only the selected anodizing voltages, *i.e.* 10 and 30 V, because these samples have been chosen, as the representative, for further microscopic and spectroscopic analyses. The surface appearance of a sample in the as-delivered state was characterized by significant unevenness and a pattern of scratches resulting from the rolling of the sheet from which disc samples were cut (Fig. 1). Chemical etching did not involve significant changes, apart from showing the grain boundaries (Fig. 1). A common feature of all anodized samples was the variable color, which depended on the anodizing voltage. At the lowest of the applied voltages (5 V), a light yellow color was observed, which became more intense after increasing the voltage to 10 V (Fig. 1). At 15 V the color started to change to light blue to achieve a distinct blue tint at 20 V. Increasing the anodizing voltage to 30 V resulted in a clear saturation of the blue color (Fig. 1).

**Fig. 2:**
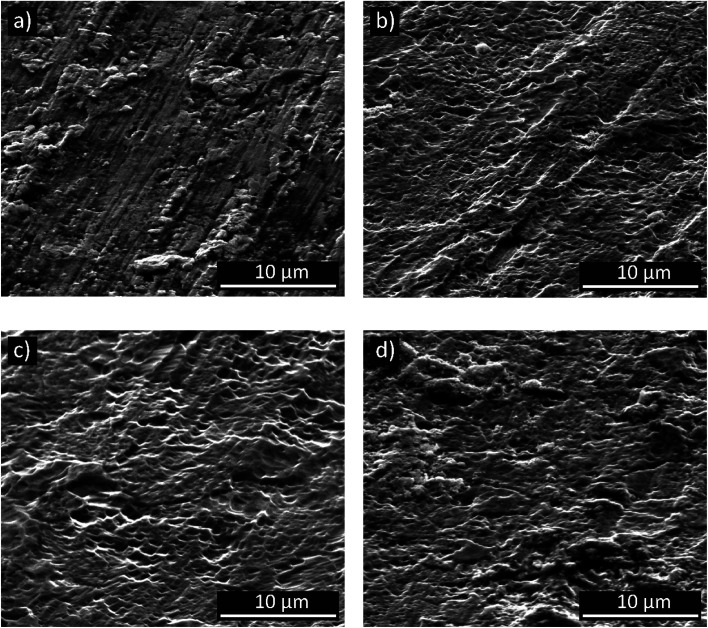
Coarse SEM microphotographs of titanium samples after: degreasing (a), degreasing and etching (b) and after anodic polarization in a DES bath at 10 (c) and 30 V (d) for 10 min at 60 °C.

Fig. 2a presents a SEM microphotograph of the as-delivered titanium sample after only degreasing step. For this surface, parallel scratches derived from rolling operation were observed. In addition, the surface was characterized by significant heterogeneity and protruding pieces of material, probably the result of plastic deformation.

A clear change in morphology was observed only after the etching of titanium (Fig. 2b). The use of an etching solution, composed of HNO_3_ and HF, activated titanium and revealed its microstructure. The surface of the grains was also etched, which in places led to the formation of micropore-like structures (Fig. 2b). As the anodizing voltage was 10 V (Fig. 2c), the said “porosity” did persist, but after anodizing at 30 V (Fig. 2d) it ceased to be the dominant feature. However, the grain boundaries were still visible (Fig. 2d).

**Fig. 3:**
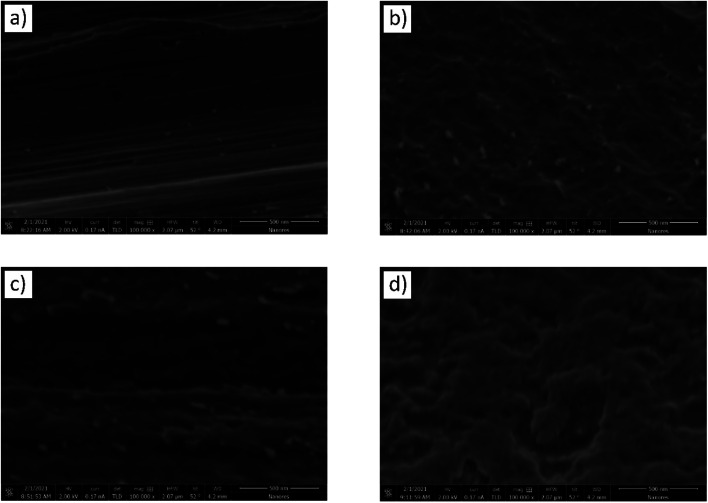
HR-SEM microphotographs of titanium samples after: degreasing (a), degreasing and etching (b) and after anodic polarization in a DES bath at 10 (c) and 30 V (d) for 10 min at 60 °C.

**Fig. 4:**
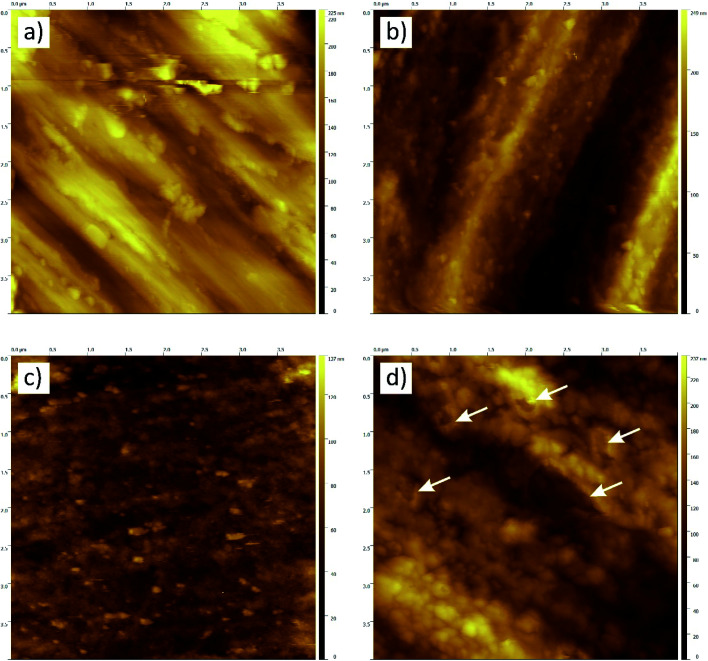
AFM topography of titanium samples after: degreasing (a), degreasing and etching (b) and after anodic polarization in a DES bath at 10 (c) and 30 V (d) for 10 min at 60 °C.

Due to the expected very small thickness of anodic layers, the samples were further subjected to a high-resolution (HR-SEM) and atomic force (AFM) microscopy, which are definitely better to evaluate morphology and topography of thin films. HR-SEM and AFM analyses confirmed formation of the oxide layer and a clear change in a layer morphology when the anodizing voltage has been increased from 10 to 30 V (Fig. 3 and 4). Moreover, the observed layer morphology confirms that anodization in proposed DES is gentle and takes place in a completely different way than when DES solvent is used only as an additive to the anodizing bath.^23^

Titanium surface after only degreasing was characterized by a parallel arrangement of fine scratches (Fig. 3a) with some irregularities revealed in the AFM microscope photo (Fig. 4a) and surface area roughness, *S*_a_, equal to 20.8 nm. Chemical etching caused these scratches to disappear and created more wavy surface (Fig. 3b). The observed surface morphology was granular and, due to higher *S*_a_ (34.5 nm), very likely that this texture reflected titanium grains, as evidenced by AFM in Fig. 4b. After anodizing at 10 V this wavy surface was somewhat “smoothed” (Fig. 3c) but still remained granular with lower *S*_a_ = 10.9 nm (AFM photo in Fig. 4c). After increasing the anodizing voltage to 30 V, the formation of a probably thicker and quite uneven oxide layer was observed (Fig. 3d). This anodic layer was characterized by a distinct and granular morphology (Fig. 4d), but the average size of the nodules together with *S*_a_ = 28.3 nm were definitely higher than those at 10 V. The topographic map of this layer also revealed some porous structures, clearly visible in the upper part of Fig. 4d (white arrows) and confirms earlier conclusions from microscopic observations Fig. 3d.

The results of microscopic analyzes: optical, scanning electron and atomic force indicate the formation of an oxide layer on the surface of technical grade titanium in the process of its anodic polarization. The scanning electron microscopy (SEM) technique used in conjunction with energy dispersion spectroscopy microanalysis (EDS), however, turned out to be insufficient. Therefore, it was decided to use X-ray photoelectron spectroscopy (XPS), which is predisposed to study the chemical composition and morphology of *e.g.* very thin passive layers on metals.

### 3.2. Surface chemistry of bare and anodized titanium

Native passive layers on titanium are of low thickness – usually of a few nm. Those made artificially, in the anodizing process, are definitely thicker – tens of nanometers. Due to the low layer thickness in combination with the sampling depth in the XPS (several nanometers) technique, the result of the XPS analysis of such layers can be largely “distorted” by the outermost layer of contamination (adventitious) carbon. Therefore, first XPS analyzes were performed both on the surface in the as-delivered state (*i.e.* without Ar^+^ sputter cleaning) and then after short and gentle Ar^+^ sputter clearing. This gentle ion bombardment (90 s, 1 keV, 1.5 μA cm^−2^) allowed to get rid of this outermost layer of carbon, reveal the composition of the titanium anodic layer and at the same time avoid photoreduction of titanium.

**Fig. 5:**
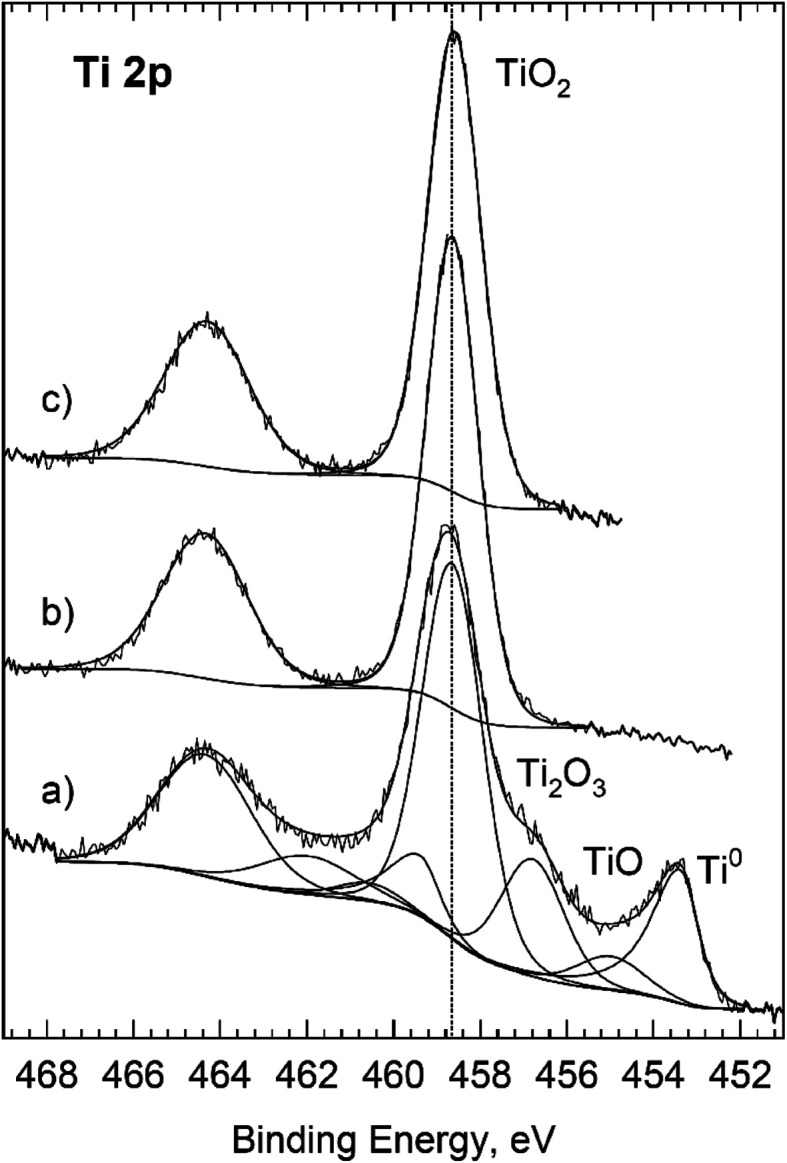
XPS Ti 2p spectra for titanium samples: etched (a) and anodized at 10 (b) and 30 V (c). Surface was Ar^+^ sputter cleaned before this analysis.

Table 1 shows the surface composition of titanium degreased and etched, and additionally anodically polarized. The surfaces of all analyzed titanium samples were covered with a layer of its natural oxides of various thicknesses, and the most outer surface was made of adsorbed carbon (adventitious carbon). Carbon, present on the surfaces of the samples in significant amounts, was a typical contamination carbon. Its quantity decreased very quickly after Ar^+^ sputtering, from *ca.* 21 at% to *ca.* 4 and 7 at% (Table 1). The structure of carbon bonds (spectra not included) in both anodized samples, *i.e.* 10 V and 30 V was similar: C–C/CH (56% and 50%), C–O (18 and 20%), C=O (8 and 10) and COO (18 and 20%), respectively. It is worth noting that after Ar^+^ cleaning the share of COO/COO–R groups still remained high, and even grew to 27% (at 10 V). Most likely it was an insufficiently rinsed, adsorbed residue of oxalic acid or choline dihydrogencitrate (spectra not included). On the surface of the reference Ti sample (degreased and etched) the share of COO groups was lower (*ca.* 9%), and after Ar^+^ cleaning (under the same parameters) it dropped to zero. Slightly higher carbon content on the surface of titanium anodized at 30 V could result from a greater surface roughness, and thus lower effectiveness of the Ar^+^ ion bombardment. After Ar^+^ cleaning of the surface of anodized samples, the ratio O : Ti = 2 : 1, pointing to the stoichiometric TiO_2_. Additionally, it was confirmed by the deconvolution of high-resolution Ti 2p spectra (Fig. 5b and c). Estimated bond energy for the Ti 2p_3/2_ and Ti 2p_1/2_ doublet was, respectively, 458.64 and 464.34 eV, which is characteristic of bulk TiO_2_. In the envelope of Ti 2p spectrum neither the Ti(0) component derived from the metallic substrate nor the Ti component in the lower oxidation states, *i.e.* Ti^3+^ or Ti^2+^, was noticed. The absence of a metallic component also meant that the thickness of the oxide layer was greater than approx. 4 nm, because as is commonly accepted sampling depth in XPS is 3*λ*_IMFP_, as in the case of Ti 2p photoelectrons in TiO_2_ means thickness of 3 × 1.2 nm = 3.6 nm.^29^

**Table 1:** Chemical composition (at%) of titanium surface after chemical etching and after anodic polarization in a DES bath composed of choline dihydrogencitrate and oxalic acid at 10 and 30 V for 10 min at 60 °C^a^

Sample	Spectral line and atomic ratio					
	Ti 2p	O 1s	C 1s	C : Ti	O : Ti	C : O
Etched Ti_(as-received)_	24.94	47.36	27.71	1.11	1.90	0.59
Etched Ti_(Ar^+^)_	39.03	58.08	2.89	0.07	1.49	0.05
10 V_(as-received)_	22.76	56.59	20.65	0.91	2.49	0.36
10 V_(Ar^+^)_	31.45	64.9	3.65	0.12	2.06	0.06
30 V_(as-received)_	21.73	57.63	20.63	0.95	2.65	0.36
30 V_(Ar^+^)_	29.61	63.56	6.83	0.23	2.15	0.11

a _(as-received)_ – sample without Ar^+^ sputter cleaning, _(Ar^+^)_ – surface of the sample was sputter cleaned with Ar^+^.

A more detailed morphology of the oxide layers produced on titanium by the anodic polarization method as well as for titanium native oxides layers (as reference), was modeled using QUASES “Analyze” software.^30^

**Fig. 6:**
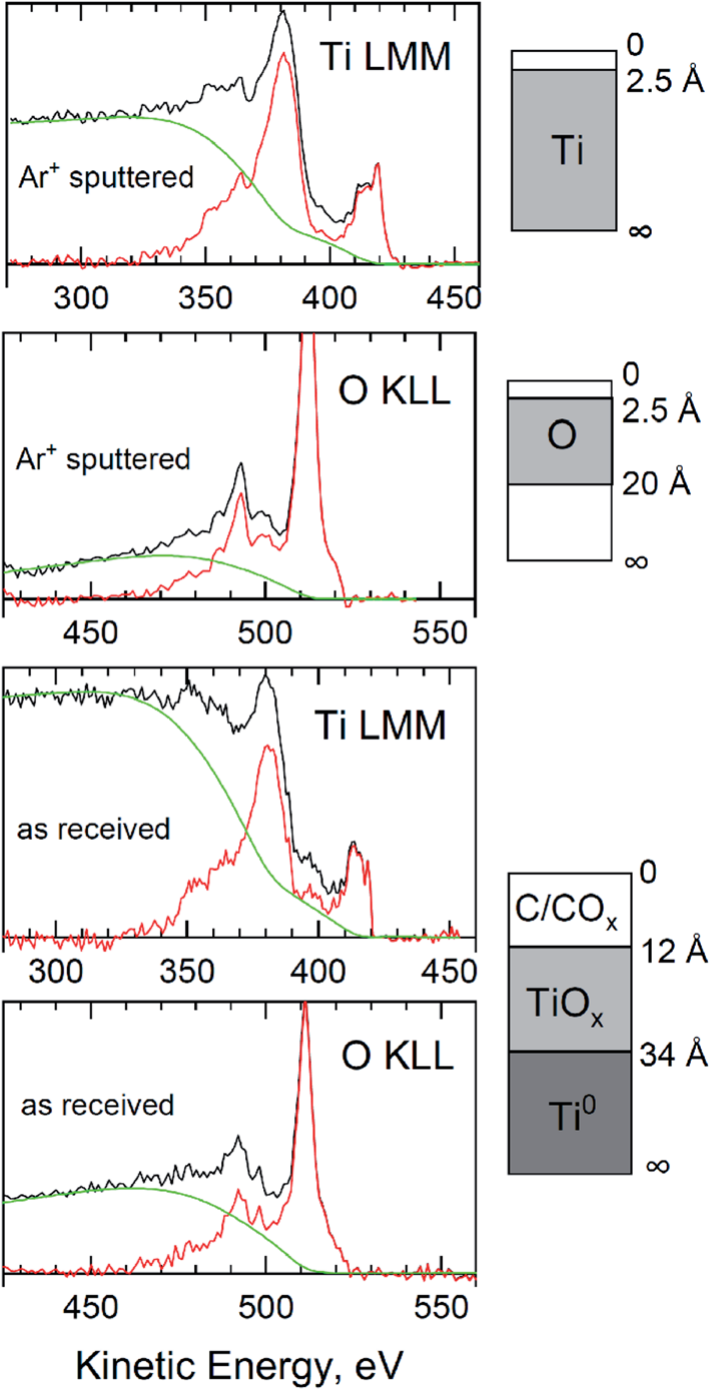
QUASES™ “Analyze” modeled peaks from titanium surface (after chemical etching) with a native oxides layer.

Overlayer thicknesses from deposited adventitious carbon, for Ti native layer, were first determined by analysis of the Ti LMM and O KLL regions (Fig. 6 “as received”) using the “Analyze” program and Mg Kα excitation. Then, the thickness of the oxide layer was determined using the O KLL region, but after removing the contamination carbon from the surface (Fig. 6 “Ar^+^ sputtered”). All spectra in Fig. 6 show a good overlap of the energy loss cross-section with the extrinsic loss portion of the scans. From “Analyze”, assuming “burried layer model”, a carbon overlayer of 1.2 nm and an overall film thickness of 3.4 nm were determined. At this thickness of the passive layer, not only the dominant peak was visible in the high resolution Ti 2p spectrum of TiO_2_, but also metallic Ti (from the substrate), TiO and Ti_2_O_3_ in shares respectively: 25, 55.5, 1.3 and 18.2% (Fig. 5). The procedures described in the works of Biesinger *et al.*^31^ were used in the deconvolution of the Ti 2p spectra.

**Fig. 7:**
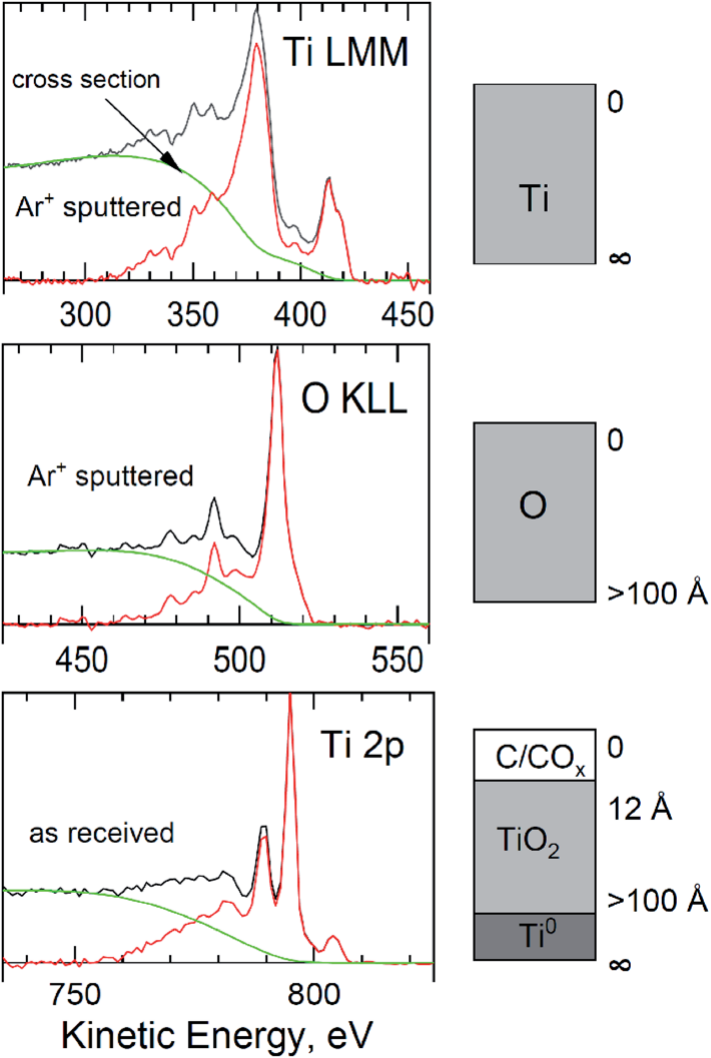
QUASES™ “Analyze” modelled peaks from titanium surface anodized at 10 V for 10 min at 60 °C.

For the oxide layer formed on titanium during polarization at 10 V, a similar analytical procedure was used with QUASES “Analyze” software. In the first step, the thickness of the contamination carbon layer was determined by analyzing the Ti 2p region (and Ti LMM, but spectrum not included). Based on the results obtained with the “Analyze” program, the thickness of adventitious carbon layer was calculated as 1.2 nm for the as received sample (Fig. 7). However, it turned out that it was impossible to accurately estimate the thickness of the oxide layer. Because a good overlap of the energy loss cross-section with the extrinsic loss portion of the scans were obtained for all layers with modeled thicknesses above 10 nm. Even if contamination carbon was removed from the surface, the thickness of the oxide layer was still higher than the max of *ca.* 10 nm (with Mg Kα excitation beam).

### 3.3. The effect anodic layer on the corrosion resistance of titanium

#### 3.3.1. dc corrosion study in 0.05 M NaCl solution – instantaneous corrosion rate

**Fig. 8:**
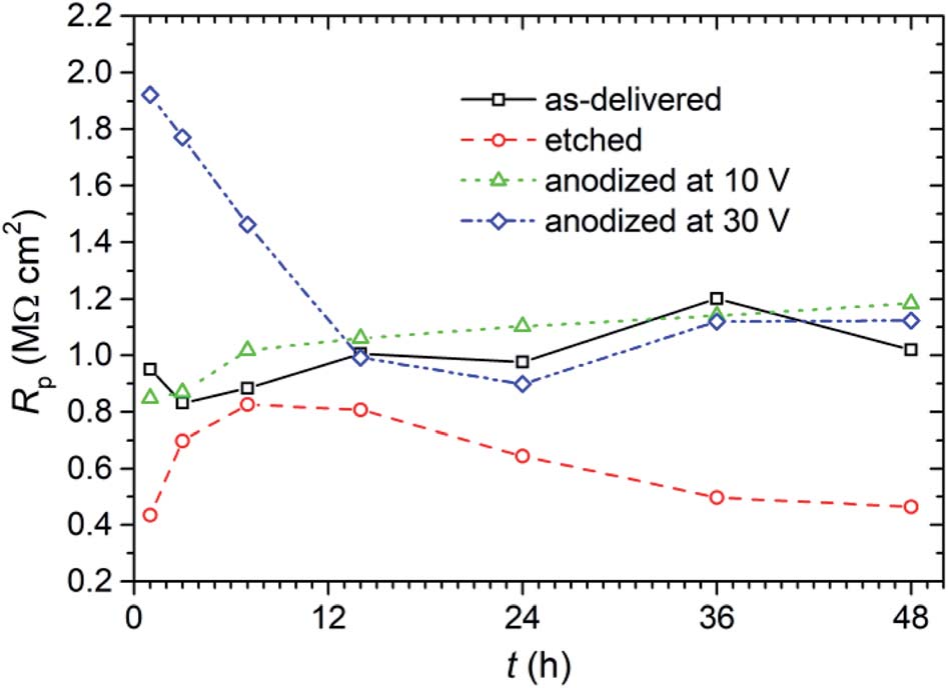
Polarization resistance (*R*_p_) measured in 0.05 mol dm^−3^ solution of NaCl during 48 h exposure of titanium samples with anodic layer and the reference samples.

The effect of anodizing process in ChCit : oxalic acid eutectic solvent on the corrosion resistance of titanium was assessed by linear polarization resistance (LPR) and polarization curves. First the non-destructive LPR technique has been applied, that enabled estimating and monitoring the instantaneous corrosion rate very quickly. The resulting polarization resistance (*R*_p_) as a function of exposure time of the samples in 0.05 mol dm^−3^ solution of NaCl is presented in Fig. 8.

**Fig. 9:**
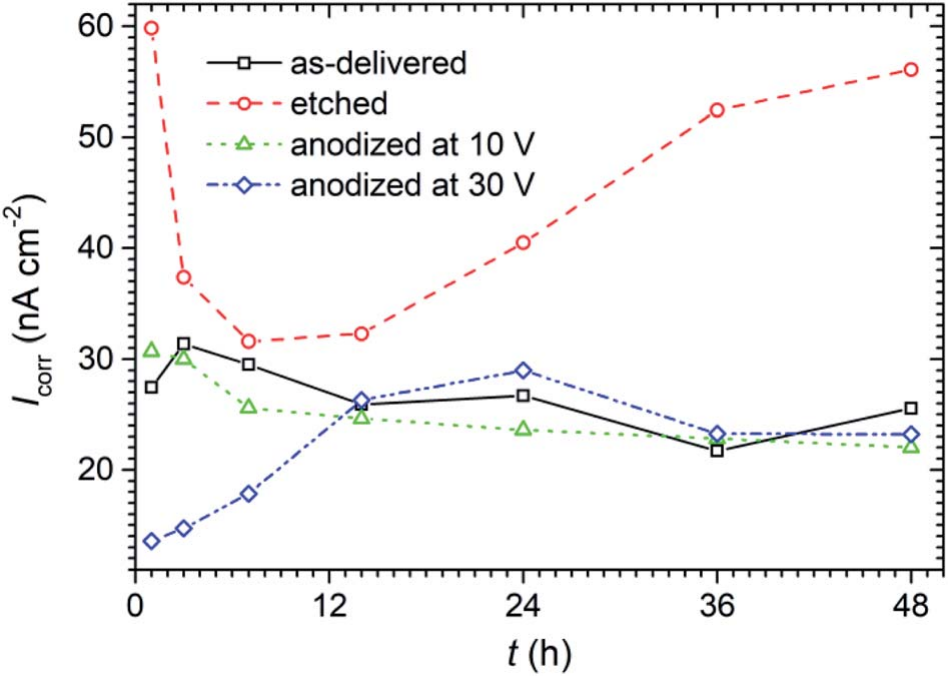
Corrosion current density (*I*_corr_) values calculated from LPR method for titanium samples with anodic layer and the reference samples exposed for 48 h in 0.05 mol dm^−3^ solution of NaCl.

From LPR method the values of corrosion current density (*I*_corr_) were calculated using Stern–Geary equation (assuming *β*_c_ and *β*_a_ equal to 120 mV dec^−1^). The evolution of this parameter as a function of exposure time in 0.05 mol dm^−3^ solution of NaCl during exposure is presented in Fig. 9.

**Fig. 10:**
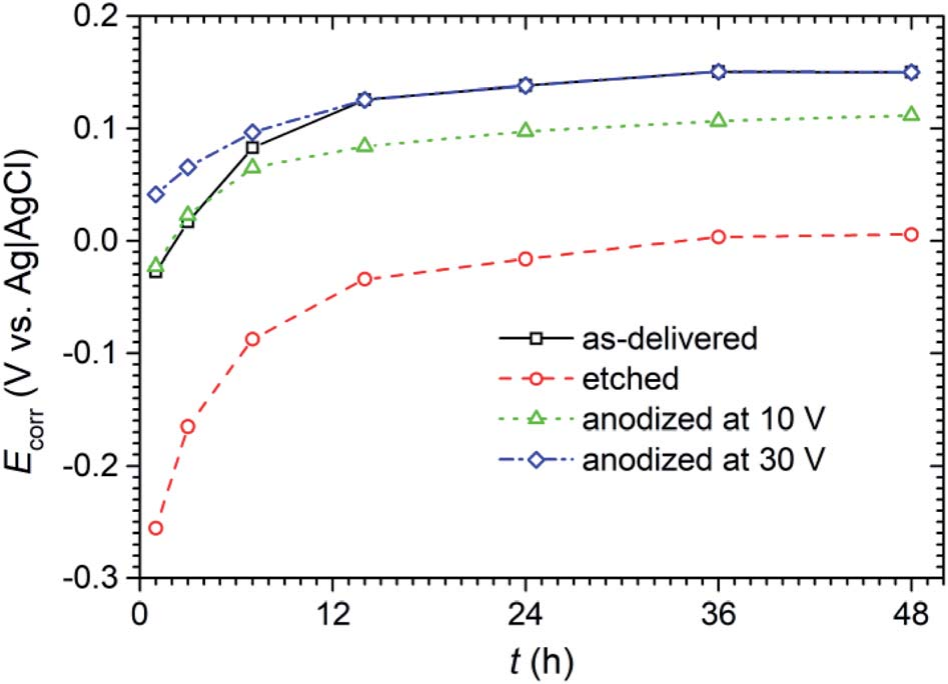
Corrosion potential (*E*_corr_) determined from LPR measurements in 0.05 mol dm^−3^ solution of NaCl over 48 h of exposure of titanium samples with anodic layer and the reference samples.

During the LPR measurements, attention was also paid to monitor the corrosion potential (*E*_corr_) of the anodized titanium in NaCl solution (Fig. 10). Although it is not a parameter that determines corrosion resistance, it gives some information about the state of the surface of the tested material and its interaction with the corrosive environment.

**Fig. 11:**
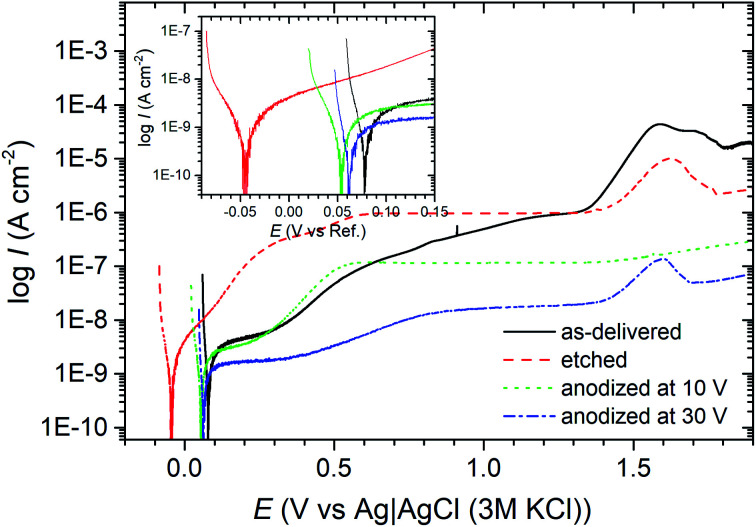
Polarization curves of titanium samples with anodic layers, and the reference samples, recorded after 48 h of exposure in 0.05 mol dm^−3^ solution of NaCl.

Finally, after 48 h of exposure of the samples in 0.05 mol dm^−3^ solution of NaCl, polarization curves have been registered in a potentiodynamic mode (Fig. 11). The curves obtained for the samples etched in HNO_3_ and HF show that chemical etching activated the surface, which caused a noticeable increase in the corrosion current density value and a shift towards negative values (by about 60 mV) of a cathodic–anodic transition potential compared to this potential for of samples “as-delivered” (Fig. 11). Chemical etching affected also the course of anodic branch. The slope of this curve (Fig. 11) was definitely smaller than all other samples, which probably indicates worse barrier properties (possibly: a thin, leaky passive layer created on the etched titanium surface).

Potentiodynamic polarization measurements have clearly shown that subjecting titanium to polarization in EDS (ChCit : oxalic acid DES solvent) increases its corrosion resistance. In Fig. 11 it is well visible, that the corrosion current densities of titanium anodized at 10 and 30 V were lower than that of non-treated titanium. It is also worth emphasizing the fact that the current densities measured in the passive range at the anodic branch were also clearly smaller, especially for the anodizing voltage of 30 V (Fig. 11). That may suggest the presence of a dense/coarser oxide layer on the surface of this sample.

#### 3.3.2. Electrochemical impedance spectroscopy (EIS) in 0.05 M NaCl solution

**Fig. 12:**
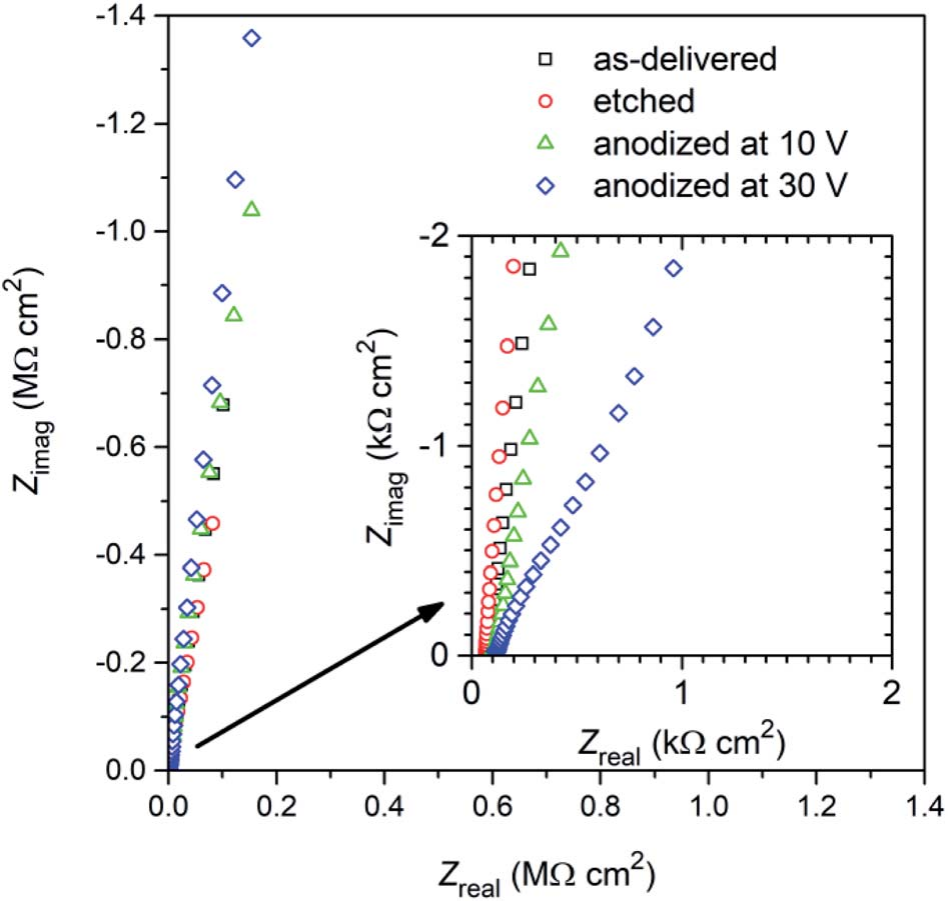
Nyquist plot of the impedance spectra recorded after 48 hours exposure in deaerated 0.05 mol dm^−3^ NaCl solution of titanium samples: after degreasing (“as-delivered”), degreasing and etching (“etched”) and after additional anodic polarization at 10 and 30 V at 60 °C for 10 min in DES anodizing bath.

**Fig. 13:**
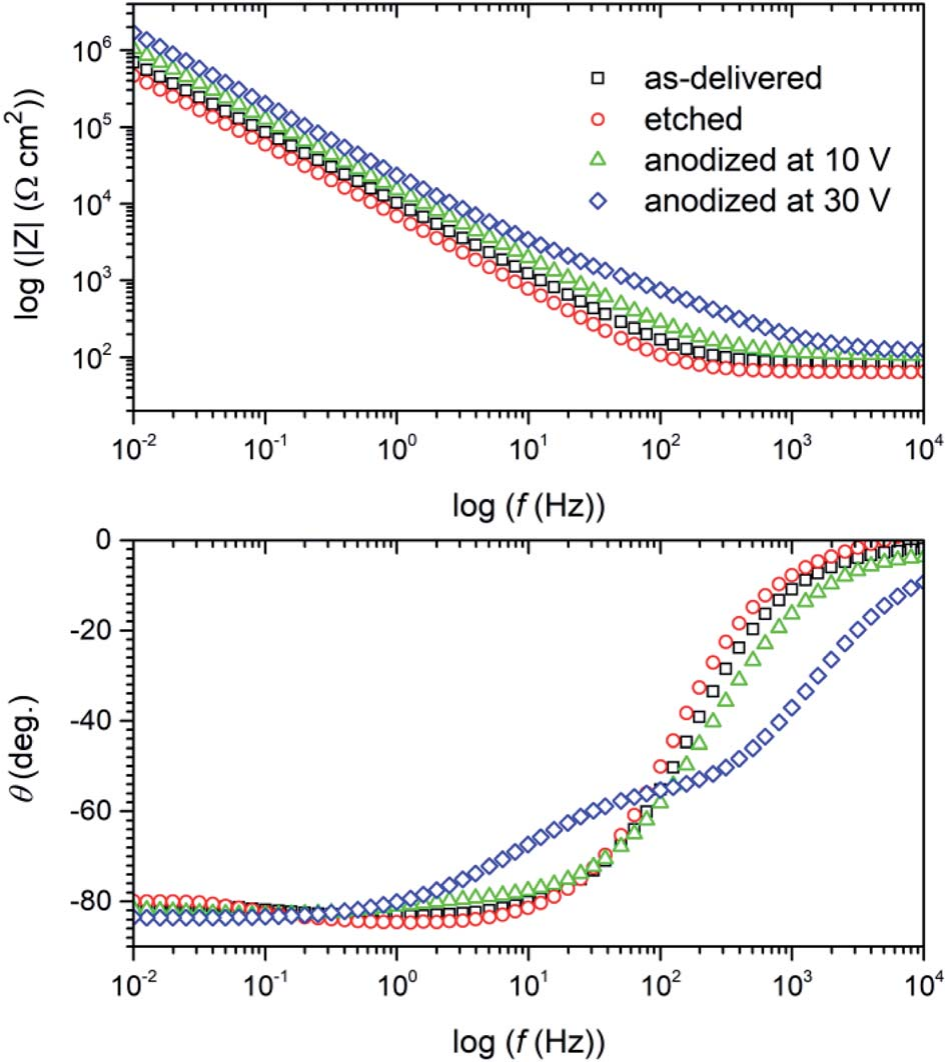
Bode plot of the impedance spectra recorded after 48 hours exposure in deaerated 0.05 mol dm^−3^ NaCl solution of titanium samples: after degreasing (“as-delivered”), degreasing and etching (“etched”) and after additional anodic polarization at 10 and 30 V at 60 °C for 10 min in DES anodizing bath.

Impedance spectra have been collected after 48 h of exposure of titanium samples in deaerated 0.05 mol dm^−3^ NaCl solution. They have been presented in the Nyquist (Fig. 12) and in the Bode (Fig. 13) representations to better justify the choice of the physical model for fitting procedure.

A cursory analysis of the Nyquist plot (Fig. 12) seemingly suggests a similar corrosion behavior of all titanium samples. However, the spectra also indicates the existence of a time constant in the range of medium frequencies (figure inset in Fig. 12), especially in the impedance spectrum of titanium anodized at 30 V. Attention is drawn to the very high values of the imaginary part of impedance reaching values exceeding 1 MΩ cm^2^. It can be assumed that this is due to an oxide layer with very good barrier properties, which translates into high corrosion resistance of the material, *i.e.* in the Bode representation of impedance spectra (Fig. 13) we observe significant increase of the impedance modulus at 0.01 Hz (|*Z*|_0.01 Hz_) after anodization. Another change, which is undoubtedly related to the formation of anodic oxide layer, was the appearance of two, although not clearly separated, phase angle (*θ*) maxima at *ca.* 400 Hz and several Hz (Fig. 13) for sample anodized at 30 V.

Moreover, the phase angle (*θ*) did not decrease even at the lowest frequencies (Fig. 13). This would confirm the existence of oxide layer barriers, because otherwise, *i.e.* if the titanium underwent active corrosion, the imaginary part of impedance would decrease (*Z*_imag_ in Nyquist plot – Fig. 12) and we would observe a decrease in the value of |*Z*| in this frequency range.

**Fig. 14:**
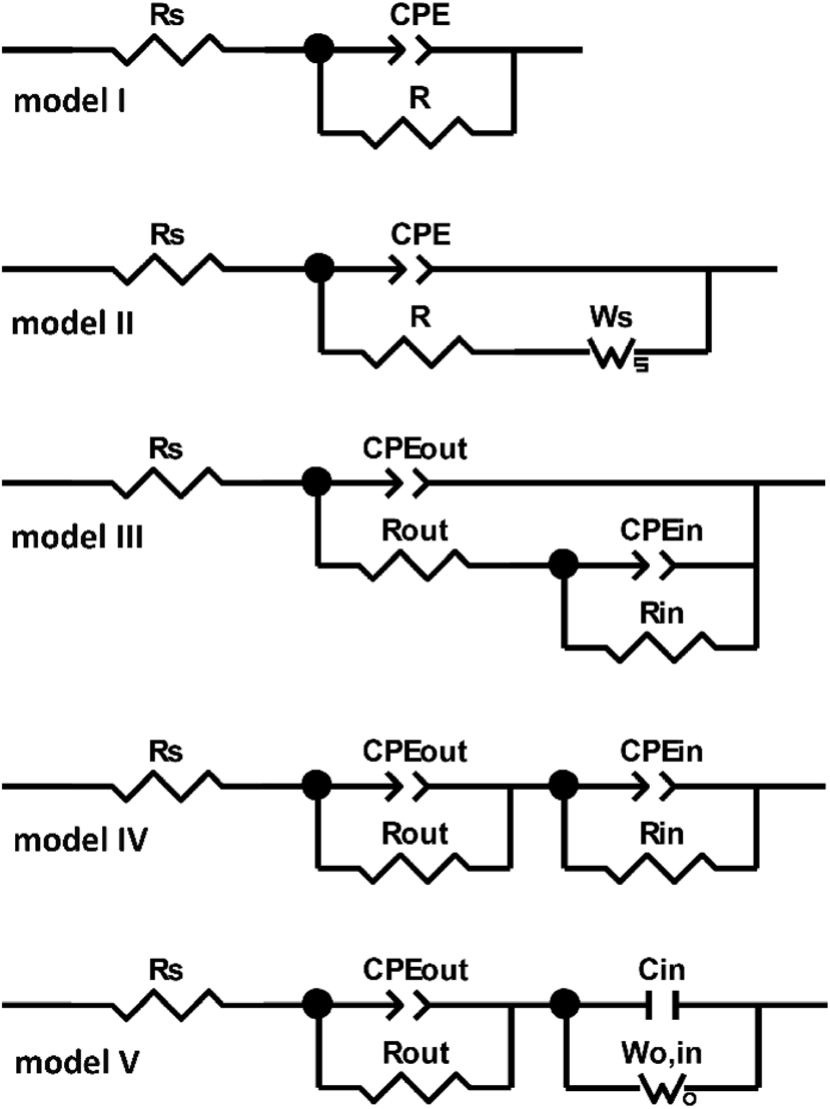
Electric equivalent circuits used for fitting the impedance spectra of titanium samples: after degreasing (“as-delivered”), degreasing and etching (“etched”) and after additional anodic polarization at 10 and 30 V at 60 °C for 10 min in DES anodizing bath, recorded after 48 hours exposure in deaerated 0.05 mol dm^−3^ NaCl solution.

There are many electric equivalent circuits in the literature to model the corrosion resistance of titanium. The first of them is the Randles-type circuit (model I and II in Fig. 14), which only takes into account the resistance of electrolytic solution and the polarization resistance connected in parallel with capacitance (alternatively modeled with a constant phase element – CPE).^32,33^ The impedance of the CPE is defined by eqn (1), where: *T* is a time constant parameter (Ω^−1^ cm^−2^ s^−*P*^), *ω* is the angular frequency of the ac signal and *P* is the CPE exponent.

1 *Z*_CPE_ = *T*^−1^(j*ω*)^−*P*^

The second-type circuits are more complex (model III–V in Fig. 14). The unquestionable advantage of the two-time constant circuits is the ability to model the morphology of oxide film.^33,34^ J. Pan *et al.*^34^ used model III (Fig. 14) to simulate a two-layer structure of titanium oxide film which was composed of thin inner layer with excellent barrier properties and a relatively porous outer layer.

Fitting with one-time constant circuit from Fig. 14 gave satisfactory results but only for “as-delivered” and “etched” samples. The determined electrical quantities *R*_s_ and CPE had very low residual errors (0.07–0.58%). Only for *R* the error was greater and amounted to 9.9–12.1%, which was related to the deterioration of the lowest frequency alignment – possibly due to diffusion through the oxide layer. The physical meaning of the elements used in this model were as follows: *R*_s_ – the resistance of electrolytic (0.05 mol dm^−3^ NaCl) solution, *R* and CPE – the resistance and capacitance describing both the properties of passive oxide layer and faradaic process. This is an obvious simplification which can be a good reference point when optimizing the physical model. Fitting results summarized in Table 2 clearly indicate a much lower corrosion resistance (three times lower resistance, *R*) of etched sample. Apart from this observation, it is difficult to infer about the oxide layer structure because the model used does not allow it.

**Table 2:** Fitting results using model I and model II for impedance spectra recorded after 48 h exposure in deaerated 0.05 mol dm^−3^ NaCl solution of titanium samples without anodizing in DES bath^a^

Sample	Model	*R*_s_ (Ω cm^2^)	CPE-*T* (Ω^−1^ cm^−2^ s^−*P*^)	CPE-*P*	*R* (Ω cm^2^)	*W*_s_			Goodness of fit (*χ*^2^)
						*W*_s_-*R*	*W*_s_-*T*	*W*_s_-*P*	
As-delivered	Model I	82	1.80 × 10^−5^	0.926	1.03 × 10^7^	n.a.	n.a.	n.a.	1.9 × 10^−4^
	Model II	82	1.73 × 10^−5^	0.935	9.16 × 10^5^	1.12 × 10^7^	51.9	0.67	6.5 × 10^−6^
Etched	Model I	64	2.65 × 10^−5^	0.944	3.25 × 10^6^	n.a.	n.a.	n.a.	6.2 × 10^−4^
	Model II	64	2.46 × 10^−5^	0.960	2.08 × 10^5^	4.32 × 10^6^	63.9	0.61	9.4 × 10^−5^

a n.a. – not applicable in model I.

Some improvement in the fit was obtained after extending the model I with an additional Warburg (*W*_s_) element, connected in series with *R* (model II in Fig. 14), whose impedance (*Z*_*W*_s__) can be represented by eqn (2), where: *R* reflects diffusion impedance, *T* reflects time of diffusion of the particle through layer thickness, *ω* is the angular frequency of the ac signal and *P* is the *W*_s_ exponent.

2 *Z*_*W*_s__ = *R* × tanh((*IωT*)^*P*^)/(*IωT*)^*P*^

There are no major differences between the pseudocapacitances determined from both equivalent circuits (model I and II) – Table 2. However, significant differences are visible in the case of resistance values (*R*). The use of the *W*_s_ element meant that total resistance (*R* in Table 2 for model I) was distributed between the *R* and the *W*_s_-*R* parameter. Resistance, *R*, in model II decreased in the case of both samples by about an order of magnitude, while the *W*_s_-*R* parameter began to dominate and assumed values similar to those calculated for the resistance *R* in model I (Table 2). In turn, the *W*_s_-*P* parameter reached values of 0.6–0.7, which, however, differ from *P* = 0.5 for pure diffusion through a layer.

The attempt to use models I and II for anodized samples was unsuccessful. Also model III (Fig. 14) previously used by J. Pan *et al.*^34^ did not apply here despite numerous fitting attempts. Ultimately, the model IV proved to be successful in the case of titanium samples anodized at 10 V and 30 V (Fig. 14). The physical sense of the elements used in this model was as follows: *R*_s_ – the resistance of electrolytic (0.05 mol dm^−3^ NaCl) solution, *R*_out_ and CPE_out_ – the resistance and capacitance of the outer porous oxide layer (electrolyte resistance inside this porous/less dense/less compact layer), *R*_in_ and CPE_in_ – the resistance and capacitance of the inner barrier oxide layer. According to Table 3 the outer porous layer, *R*_out_, resistance was close to 1.2 kΩ cm^2^ and very similar for both anodized surfaces. However, the electrical (pseudo)capacitance of the outer layer, CPE_out_, was much smaller (probably in the order of a dozen μF cm^−2^) for the sample anodized at 30 V. Also the exponential parameter *P* of CPE_out_ for that surface was slightly higher, which could also indicate its lower heterogeneity (Table 3). The same observation applies to the pseudocapacitance of CPE_in_ for “30 V” sample. The biggest difference, however, was in the resistance of the inner layer, *R*_in_. While after anodizing at 10 V the resistance of inner layer was *ca.* 28 MΩ cm^2^, then after anodizing at 30 V, *R*_in_ was calculated of 10^13^ Ω cm^2^ (!). Of course, it is unlikely, especially since error for this element was 10^7^%, as indicated in Table 3. This prompted us to modify the equivalent circuit for this sample. *R*_in_ and CPE_in_ elements were replaced with open Warburg (*W*_o,in_) and pure capacitor (*C*_in_), respectively – model V in Fig. 14. Ultimately, a very good fit was obtained (*χ*^2^ = 1.5 × 10^−5^) and errors *ca.* 0.2–5.2%. The component values calculated from this fine model are shown in Table 4.

**Table 3:** Fitting results using model IV for impedance spectra recorded after 48 h exposure in deaerated 0.05 mol dm^−3^ NaCl solution of titanium samples after anodization at 10 and 30 V at 60 °C for 10 min in DES bath composed of choline dihydrogencitrate salt and oxalic acid

Sample	*R*_s_ (Ω cm^2^)	CPE_out_-*T* (Ω^−1^ cm^−2^ s^−*P*^)	CPE_out_-*P*	*R*_out_ (Ω cm^2^)	CPE_in_-*T* (Ω^−1^ cm^−2^ s^−*P*^)	CPE_in_-*P*	*R*_in_ (Ω cm^2^)	Goodness of fit (*χ*^2^)
Anodized at 10 V	105	2.51 × 10^−4^	0.690	1.29 × 10^3^	1.23 × 10^−5^	0.929	2.77 × 10^7^	2.4 × 10^−5^
Anodized at 30 V	111	1.88 × 10^−5^	0.729	1.16 × 10^3^	7.72 × 10^−6^	0.927	8.91 × 10^13^^a^	7.9 × 10^−5^

a 2 × 10^7^% error for this element.

**Table 4:** Fitting results using model V for impedance spectra recorded after 48 h exposure in deaerated 0.05 mol dm^−3^ NaCl solution of titanium samples after anodization at 10 and 30 V at 60 °C for 10 min in DES bath composed of choline dihydrogencitrate salt and oxalic acid

Sample	*R*_s_ (Ω cm^2^)	CPE_out_-*T* (Ω^−1^ cm^−2^ s^−*P*^)	CPE_out_-*P*	*R*_out_ (Ω cm^2^)	*C*_in_ (F cm^−2^)	*W*_o_			Goodness of fit (*χ*^2^)
						*W*_o_-*R*	*W*_o_-*T*	*W*_o_-*P*	
Anodized at 10 V	103	1.52 × 10^−4^	0.677	880^aa^	8.99 × 10^−6^	70 184^b^	0.169	0.39	7.2 × 10^−6^
Anodized at 30 V	114	1.32 × 10^−5^	0.764	826	4.38 × 10^−6^	25 836	0.059	0.43	1.5 × 10^−5^

a 21% error for this element.

b 2% error for this element.

## 4. Conclusion

• It has been shown that the technical grade titanium anodizing process can be successfully transferred to the alternative green solvent DES, composed of choline dihydrogencitrate salt–oxalic acid in 1 : 1 molar ratio.

• The change in voltage in the DES titanium anodizing process affects the color of the formed oxide layer in a similar degree to that of conventional water-bath anodizing.

• After anodizing at 10 V, the titanium surface shows a fine-grained structure. At 30 V, a coarser, rather uneven, oxide layer is formed, with significantly larger grains.

• The as-delivered titanium surface was covered with an oxide layer, the thickness of which was estimated to be *ca.* 2.2 nm (plus extra 1.2 nm of adventitious carbon in the top-layer). DES anodizing produces a homogeneous TiO_2_ layer whose thickness at a potential of 10 V exceeds 10 nm.

• As shown by XPS tests, the stoichiometric TiO_2_, regardless of the applied voltage in the anodizing process, was practically the only component of the oxide layer produced on titanium in the DES bath.

• The potentiodynamic polarization technique showed that the passive layers produced in anodizing process of titanium in the DES bath exhibit at least one order of magnitude greater corrosion resistance compared to the layers present on titanium as delivered.

• Electrochemical impedance spectroscopy (EIS) allowed to describe the layers formed in the anodizing process using a two-layer model. The barrier properties correspond to the inner layer formed directly on the surface of metallic titanium, the maximum resistance of which was 2.8 MΩ cm^2^. The resulting porous outer layer has a much lower resistance which ranges from 830 Ω cm^2^ to 1300 Ω cm^2^. The native oxide layer on the Grade 2 titanium surface showed a polarization resistance of approx. 1 MΩ cm^2^. The choice of the best electrical equivalent circuit allowed us to conclude that the phenomenon of diffusion (mass transport through the passive layer) has a dominant share in this resistance.

## Author contributions

Juliusz Winiarski, 45% share – conceptualization; methodology; investigation; project administration; supervision; resources; funding acquisition; writing – original draft; Anna Niciejewska, 10% share – investigation; visualization; writing – original draft; Monika Górnik, 5% share – investigation; Jakub Jakubowski, 5% share – investigation; resources; writing – original draft; Włodzimierz Tylus, 20% share – investigation; methodology; resources; writing – original draft; Bogdan Szczygieł, 15% share – resources; writing – review & editing.

## Conflicts of interest

Authors declare no conflict of interest.

## Supplementary Material
